# Building a better society: The Vital role of Family's social values in creating a culture of giving in young Children's minds

**DOI:** 10.1016/j.heliyon.2024.e29208

**Published:** 2024-04-03

**Authors:** Walaa Elsayed

**Affiliations:** a College of Humanities and Sciences, Ajman University, Ajman, United Arab Emirates; b Humanities and Social Sciences Research Center (HSSRC), Ajman, United Arab Emirates

**Keywords:** Families, Social values, Children, Culture of giving

## Abstract

This study aimed to investigate the role of families in instilling social values that enhance children's awareness of the culture of giving. A descriptive research design was used, and a sample survey method was employed, with 174 children participating. Results showed that the family's role in instilling social values was rated as moderate (weighted relative weight of 61.97%), with a total weight of 9705. The study also found that children face difficulties in adhering to the encouraging social values of volunteering. Specifically, the difficulties that children face were rated as moderate (weighted relative weight of 61.58%), with a total weight of 4822. One of the most important of these difficulties was the frequent family disputes between a child's parents that do not give them a way to practice volunteering. Besides, the families of some children are not interested in explaining the nature and mechanism of applying the encouraging social values of giving. Furthermore, the study revealed statistically significant differences in children's awareness of social values based on gender, age, educational stage, parents' educational level, and family cultural level. One of the study's key recommendations is to activate the role of families and educational institutions in organizing field visits for children to participate in voluntary and humanitarian programs. This would create constructive communication between children and vulnerable groups in society, such as orphans and the elderly, and deepen children's sense of the existence of these categories within society and the importance of providing support and assistance to them. Overall, this study highlighted the crucial role of families in instilling positive social values in children, which is essential for building a compassionate and committed future generation capable of giving back to society in various fields, particularly in human services.

## 1 Introduction

The family is recognized as the fundamental unit of society, playing a crucial role in the socialization and development of children [1]. Childhood is a critical period that significantly influences an individual's personality, values, and moral principles, with lasting effects on their future stages of life [2]. It is important to highlight that childhood represents a substantial portion of the global population pyramid, making it a driving force behind societal development. Furthermore, childhood is associated with productivity, generosity, and creativity, which contribute to the advancement and prosperity of societies [3].

Education and socialization are areas of significant competition among nations, as they recognize the importance of instilling positive social values in children. These values are essential for building a positive human personality and preserving the religious, cultural, and social fabric of a society [4]. By imparting virtuous values to children, societies aim to equip them with the necessary tools to serve humanity limitlessly [5]. Education and socialization serve as mechanisms for shaping the behaviors, customs, and societal traditions that become ingrained in the lives of individuals. Societies that possess virtuous values and a strong identity are more likely to thrive in the modern era [6].

Numerous studies, including those by Pérez-Fuentes 2019, Twito 2020, and Ikhwan 2019, have indicated that social values play a crucial role in shaping children's personalities and instilling positive moral attributes that enhance their human traits, enabling them to become effective, productive, and committed members of society in the future [7]. These studies have demonstrated that values serve as the foundation for developing an individual's personality in a proper manner, aligned with the cultural norms of society [8], and promote essential human aspects such as affection, respect, mercy, justice, and social solidarity within society [9].

Besides, Barbera 2020, Elsayed 2021, Vogl 2020, and Anggadwita 2019 studies support the idea that the proper social upbringing of a child by their parents is crucial in consolidating and educating them about social values and how to effectively apply them through realistic educational and training situations that practice good social and behavioral values [10]. According to their studies, the family serves as the foundation for a child's life [11]. When parents instill human traits that reflect positive social values for children to abide by Ref. [12], it creates a strong shield against extremist ideas or abnormal intellectual and behavioral trends [13].

Other studies have examined the concept of social values. For instance, Kenter 2015, Ives 2019, and Rasoolimanesh 2020 found that social values encompass desirable characteristics or qualities that individuals and groups should uphold [14]. These values are influenced by the prevailing culture within the community, both at the local and global levels [15]. Examples of such values include tolerance, truth, strength, justice, honesty, boldness, cooperation, and altruism [16]. Similarly, studies by Lake 2021, Torbica 2020, de Vries 2018, and Boone 2020 have emphasized the role of social values in upholding social order [17] and ensuring stability within society [18]. These values are essential as they cultivate a sense of humanity and preserve the desirable moral standards within a community [19]. Without such values, the fabric of society would be compromised, and the principles that promote a harmonious coexistence would be undermined [20]. Litina 2016, Huggins 2015, Rahayu 2020, and Kronenberg 2019 have suggested that the language of values plays a crucial role in shaping individuals' behavior [21], aiding them in distinguishing between what is permissible and forbidden [22], as well as discerning right from wrong [23] and good from bad ([24]24. Arikan 2015, Agyabeng-Mensah 2020, and Nahuelhual 2016 have underscored the significance of positive values, including tolerance, honesty, and courage [25], as well as loyalty, responsibility, and a sense of belonging, in shaping an individual's interactions and behaviors towards others [26]. These values are acquired from the surrounding society in which one resides [27]. Eriksson 2019, Morris 2016, and Gould 2019 proposed that social values are integral to human principles [28], with each individual establishing their own hierarchy of social values [29] that shapes their attitudes and behaviors [30]. Similarly, Bjärstig 2016, Cannas 2019, and Littlejohns 2019 discovered that although there are numerous social values shared among humans [31], each individual assigns a unique order of importance [32] and priority to these values [33]. However, studies by Pickering 2020, MacDonald 2015, Brigandt 2015, and Busch 2016 have highlighted the existence of negative values, commonly referred to as anti-social values [34]. These values encompass traits such as selfishness, disrespect, arrogance, and hatred [35]. Unfortunately, certain individuals base their behavior and actions on these values [36], posing a threat to the security and stability of society [37].

Based on the aforementioned information, this study aims to fill a gap in the literature by investigating the role of families in instilling social values related to culture of giving in children. It seeks to explore how families can shape children's understanding and appreciation of these values, which include helping others, compassion, and making positive contributions to society. The study also aims to examine the challenges children face in adhering to these values, as influenced by their families, and the potential consequences of neglecting this role. Additionally, the study aims to explore how children's awareness of these values may vary based on factors such as gender, age, parents' educational level, and cultural background. So, the study questions will address the following areas of inquiry.

⁃ **Q1:** What is the family's role in instilling social values that enhance children's awareness of the culture of giving?
⁃ **Q2:** What are the difficulties that children face in adhering to the encouraging social values of volunteering as influenced by their families?
⁃ **Q3:** Does the degree of children's awareness of the social values that entrench their culture of giving differ according to factors such as gender, age, educational stage, parents' educational level, and family cultural background?

## 2 Literature review

### 2.1 Social values concept

Social values encompass the foundations, standards, and principles that shape an individual's beliefs and judgments towards various aspects of life [38]. They reflect an individual's conscience and influence their interaction with society, fostering interest and participation in addressing societal issues. Key social values include compassion, faith, democracy, cooperation, and social solidarity [39].

Social values also represent the standards and goals that exist in societies at different stages of development, serving as a human commitment and social necessity [40]. They are integral to the human experience, as they provide a framework for evaluating individual and group behavior [41].

Moreover, social values are fundamental to the construction of societies and nations. They are closely tied to ethics and principles that guide proper human conduct. The adherence and application of social values foster love, brotherhood, and strengthen cohesion and interdependence among members of society [42].

### 2.2 Components of social values

**Fig. 1:**
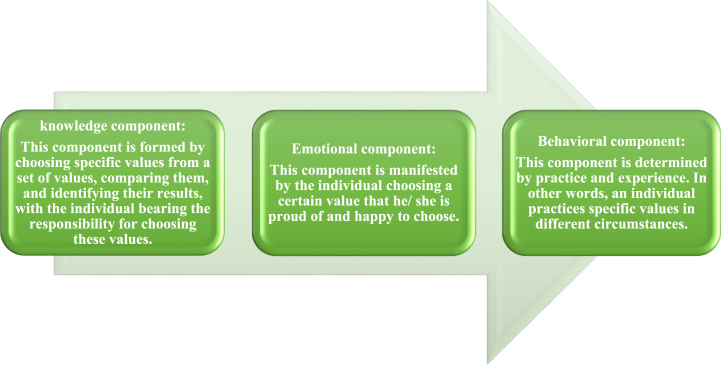
Components of social values.

Social values consist of three basic components, which are as follows:

According to Fig. 1, the components of social values are interrelated and complement each other since each component leads to the other [43]. The cognitive component leads to the emotional component, which in turn leads to the behavioral component [44].

### 2.3 Attributes of social values

- Social values are connected to human emotions and psyche, reflecting individual desires, tendencies, and emotions that vary among individuals and societies [45].
- Social values are not fixed and can change due to human interactions with the environment and changes in the surrounding circumstances [46].
- Social values are acquired through the environment and are not inherited [47].
- Social values are diverse due to the variations and diversity of human needs [48].
- Social values are subjective, appearing in people's feelings either through inclination or aversion [49].
- Social values are relative, differing from one individual to another based on their time and place [50].
- Social values are inherently linked to humanity, as they pertain to the individual or human beings and are not related to any other entities [51].

### 2.4 The importance of social values

- Social values contribute to the development of an individual's personality, helping to build a strong, mature, and coherent character [52].
- Social values empower individuals with self-control and self-regulation abilities [53].
- Social values serve as motivators, inspiring individuals to perform work and activities with excellence [54].
- Social values act as a protective shield, guiding individuals away from mistakes and providing a safeguard in life [55].
- Social values provide individuals with a sense of inner peace and tranquility [56].
- Social values contribute to a stable and balanced social life for individuals [57].
- Social values help individuals earn the love and trust of others [58].
- Social values equip individuals with the ability to coexist with contentment and satisfaction in challenging circumstances [59].

### 2.5 Social values and the basics of a good social life within society

The relationship between social values and spreading good morals: Good morals are based on societal norms and customs that are transmitted to individuals to be respected and followed [60]. Social values seek to maintain a balance of good behavior, allowing individuals to differentiate between what is good and evil, just and unjust [61]. Honesty, respect, fairness, and other social values support the dissemination of good morals in society [62].

The relationship between social values and respect for human rights: Human rights encompass principles that guarantee a decent life, promoting harmonious, respectful, tolerant, and integrated coexistence without discrimination [63]. Social values serve as common principles that regulate behavior and establish healthy coexistence between individuals [64]. Key social values that support human rights include equality, respect for human dignity regardless of religion, gender, color, and nationality, as well as the value of freedom within the boundaries of not infringing on the rights and freedoms of others [65]. The principles of democracy, such as respect for different opinions, diversity, freedom of thought, and equal rights, also play a crucial role [66].

The relationship between social values and considering society's culture: The culture of a society is built upon shared beliefs, customs, and traditions, fostering a connection to one's homeland [67]. The value of a sense of belonging, patriotism, and the connection to cultural identity are prominent social values that tie individuals to the culture of their society [68].

The relationship between social values and the consolidation of good human relations between people: Human relations are based on emotional connections developed through various forms of communication, essential for building a society for survival and continuity [69]. Positive interaction with others is crucial for individuals, as human relations are integral to their lives [70]. Social values such as love, harmony, understanding, sympathy, friendship, and altruistic assistance to others without expecting rewards support good human relations within society [71]. As social values are recognized and shape social behavior, they aim to strengthen human relations and achieve social well-being by reinforcing positive qualities in individuals during their interactions [72].

The relationship between social values and the observance of religious aspects: Religious values consist of behaviors considered correct according to monotheistic religions or individual beliefs, with individuals having the freedom to choose their own religion or belief [73]. Social values that consider religious aspects include the value of individual freedom to believe in the religion or belief system of their choice, as well as embracing virtues and qualities considered correct in their religion, such as charity, social solidarity, helping others, and love for goodness [74].

The relationship between social values and the integrity of the family entity in society: The family serves as a social bond that unites members through kinship, marriage, or adoption [75]. It involves the establishment of marriage and the care and provision for children by parents [76]. The family plays a role in instilling recognized family values through principles, beliefs, and customs passed down from generation to generation [77]. Social values that enhance family cohesion include brotherhood, unity, love, kindness, and social support among family members [78].

### 2.6 Characteristics of the Process of Instilling Good Social Values in Children's minds

**Fig. 2:**
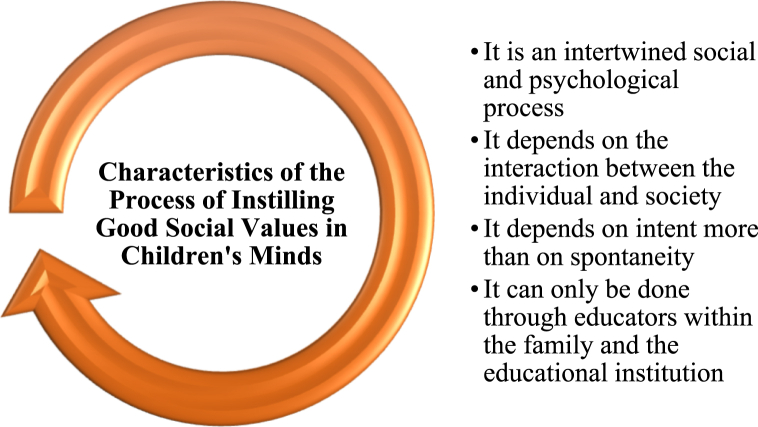
Characteristics of the process of instilling good social values in Children's minds.

According to Fig. 2, the process of instilling social values is characterized by an intertwined social and psychological process that relies on the interaction between individuals and society, with intent playing a more significant role than spontaneity [79]. This process is primarily carried out by educators within the family and educational institutions [80]. The research suggests that instilling social values in children is a delicate and crucial process, requiring individuals with the skills to interact and guide children, as well as creative abilities to positively shape their personalities within society [81].

### 2.7 Social values and children's practice of human services

Social values and human services are interconnected, with individuals who adhere to social values being more inclined to give their time, effort, and resources for the benefit of others [82]. Social values play a foundational role in shaping positive and humane aspects of the mind and conscience [83]. Values can be categorized into basic governing values, such as brotherhood, honesty, sincerity, modesty, patience, courage, and altruism, as well as complementary sub-values, including generosity, good faith, permission, asceticism, sacrifice for the sake of others, and efforts to strengthen social solidarity [84].

The participation of children in human services reflects their adherence to values such as goodness, mercy, and solidarity, and it is a significant indicator of their social well-being [85]. Engaging in human services fosters cooperation, loyalty, and the pursuit of good in society, making it crucial to instill a culture of volunteerism in children [86]. Cultivating a generation of children who embrace benevolence can contribute to the future development and growth of society [87].

### 2.8 Social values that encourage children to practice a culture of giving

Honesty: Honesty is a social value that appears in daily dealings in society, starting with the family and extending to wider society [88]. It is a character trait that involves telling the truth and being straightforward in one's actions and words. Honesty is the quality of being truthful, sincere, and free from deceit, fraud, or deception. It is an important value in personal and professional relationships, as it helps build trust, mutual respect, and integrity. Being honest also means taking responsibility for one's actions and being accountable for any mistakes or wrongdoings [89].

Altruism: Altruism is a social value that involves preferring others over oneself and putting the interests of others before one's own [90]. It is a selfless concern for the well-being of others, without expecting anything in return. Altruism is often motivated by a sense of empathy or compassion for others, and it can involve acts of kindness, generosity, and self-sacrifice. Altruistic behavior can range from small gestures, such as holding the door open for someone, to significant acts of charity or philanthropy [91]. Altruism is an important value in many cultures and religions, and it is seen as a key component of human social behavior and cooperation [92].

Giving and sacrificing: Giving and sacrificing are social values that involve making one's own interests subservient to the interests of society as a whole. Giving refers to the act of freely providing resources, such as time, money, or effort, to others or to a cause. It can involve donating to a charity, volunteering at a local organization, or simply helping a neighbor in need. Sacrificing, on the other hand, refers to the act of giving up something of value, such as time, money, or comfort, for the sake of others or a greater cause. It can involve making personal sacrifices, such as giving up one's own interests or desires, in order to benefit others or to achieve a greater good. Giving and sacrificing are important social values that promote cooperation, empathy, and a sense of community, and they are often associated with acts of charity, philanthropy, and social responsibility [93].

Modesty: Modesty is a social value that plays an important role in regulating human behavior in society. It involves having a humble and unassuming attitude towards oneself, and not being excessively proud or arrogant. Modesty can manifest in various ways, such as dressing and behaving in a way that is not attention-seeking, avoiding boastful or self-promoting language, and acknowledging one's limitations or mistakes. Modesty is often associated with humility, which is a related social value that involves recognizing one's own worth and abilities without feeling superior to others. Modesty is an important value in many cultures and religions, and it is seen as a key component of good character and social harmony [94].

Cooperation and collaboration: Cooperation and collaboration are social values that play a crucial role in human communication and are essential for individuals and communities to thrive. Cooperation involves working together with others in a coordinated and mutually beneficial way, and it can involve sharing resources, knowledge, or skills. Collaboration, on the other hand, refers to a more structured and intentional form of cooperation, where individuals or groups work together to achieve a specific objective. Collaboration can involve dividing tasks, sharing responsibilities, and utilizing each other's strengths and expertise. Both cooperation and collaboration promote teamwork, mutual respect, and a sense of community, and they are important for achieving social and economic progress [95].

Social solidarity: Social solidarity is a social value that involves the members of a society complementing each other in various aspects of life, which can reduce the sources of poverty and destitution in society. It refers to the sense of unity and cohesion that exists within a group, and it is fostered through shared values, norms, and beliefs, as well as through social interactions and collective action. Social solidarity is often associated with feelings of empathy, compassion, and mutual support, and it can play an important role in promoting social stability and resilience. It is an important value in many cultures and societies, and it is seen as a key component of social harmony and well-being [96].

Respect: Respect is a social value that is highly valued by people. It is important for individuals to respect themselves and others, as it involves acknowledging and recognizing the inherent worth and dignity of all individuals, regardless of their differences. Respect can be demonstrated in various ways, such as using polite language, listening attentively, recognizing diversity, and treating others with fairness and kindness. It is an essential value in personal and professional relationships, as it helps build trust, mutual understanding, and cooperation. Respect is also a fundamental aspect of many ethical and moral codes, and it is seen as a key component of good character and social harmony [97].

Justice: Justice is a social value that involves respecting and protecting the rights and duties of individuals in a balanced and equitable manner. It is the principle of upholding what is right and fair, and ensuring that every person is treated fairly and without discrimination. Justice can manifest in various ways, such as ensuring equal access to education, healthcare, and employment opportunities, and enforcing laws and regulations that protect individual rights and freedoms. It is an essential value in many cultures and societies, as it is seen as a key component of social order and stability. Justice is closely related to other social values, such as human rights, democracy, and the rule of law [98].

Loving and accepting others: Loving and accepting others as they are is a social value that generates happiness and comfort for individuals. It involves providing support to others without expecting anything in return, as well as showing compassion, empathy, and understanding towards individuals, regardless of their differences or flaws. This value is important for recognizing and appreciating the inherent worth and dignity of each person, and treating them with respect and kindness. Embracing diversity and refraining from judgment or criticism based on differences is essential for building strong and healthy relationships, promoting social harmony, and creating a more inclusive and equitable [99].

Freedom: Freedom is a social value that refers to the ability of individuals to express themselves and act as they wish, as long as their actions do not harm others. It is the absence of external constraints or limitations, such as coercion, oppression, or undue influence, that allows individuals to exercise their rights and pursue their goals and interests freely. Freedom can manifest in various ways, such as freedom of speech, freedom of religion, freedom of movement, and freedom of assembly [100]. It is an essential value in many cultures and societies, and it is seen as a fundamental human right. However, freedom is not absolute, and it is often limited by laws, regulations, and social norms that aim to protect the rights and well-being of individuals and society as a whole [101].

Tolerance: Tolerance refers to the acceptance that all human beings are unique creatures with strengths and weaknesses. Therefore, differences will always be present, and the most important thing is to get to know them and know how to respect them. Tolerance is a social value that involves accepting and respecting the diversity of individuals and their beliefs, values, and behaviors, even if they differ from one's own. It is the ability to recognize and appreciate the worth and dignity of each person, regardless of their differences or flaws, and to treat them with respect and kindness [102]. Tolerance requires an open-minded and non-judgmental attitude towards others, and refraining from discriminatory or prejudicial behavior based on race, ethnicity, religion, gender, sexual orientation, or any other characteristic. It is an important value in personal and professional relationships, as it helps build trust, mutual understanding, and cooperation [103].

### 2.9 Reasons for the decline in the level of adherence to social values in society

Several factors contribute to the decline in the level of adherence to social values in society, including.

- Weak religious scruples among individuals [104].
- Being affected by the negatives of social media, such as Instagram, Twitter, etc., where many negative values are promoted. While modern communication networks have positives, they also have negatives [105].
- Being influenced by negative television or press programs that spread extremist ideas and are not compatible with the culture of society [106].
- The rapid development of life and the predominance of individual interests over public interests
- Lack of sufficient awareness of the feasibility and value of values in life for some.
- Pursuing passions and desires [107].
- Being influenced by bad friends who have negative qualities and character [108].

### 2.10 Ways to build and strengthen social values in the minds and souls of children

There are several ways to build and strengthen social values in the minds and souls of children, including.

- Providing a proper family upbringing, where values and morals are instilled in the children, noting that parents are primarily responsible for the socialization process [109].
- Following the method of persuasion when teaching the child about the value and training them for it, by suggesting evidence and proofs and using them to convince children of the characteristics and importance of each value [110].
- An integrated educational system that focuses on the child's psychological and mental needs alike, and seeks to guide and nurture the student's behavior continuously [111].
- The media, by making values one of its fields and one of its goals [112].
- Religious education, whether it is in the family or through guidance in places of worship, schools, or the media [113]. All of these are integrated episodes in education, construction, and training [114].
- Having a good role model in a child's life, especially in school and at home, by teachers for their students and by parents for their children [115].

### 2.11 The educational effects achieved from Children's commitment to social values that encourage participation in human services

Committing to social values that encourage participation in human services can have several educational effects on children, including.

- Effects on the child himself: Refining the child's personality and providing them with many good skills and qualities such as giving, humility, commitment, endurance, patience, cooperation, organization, good dealing with others, and sincerity [116]. This commitment can also result in a high level of motivation, a feeling of enthusiasm, a positive view towards life, and a strong sense of hope and optimism. Children can gain self-confidence and the ability to build strong positive relationships with others while taking advantage of their free time in useful activities that benefit them and others [117].
- Effects on society: Strengthening the principle of citizenship among children, investing the energies of a new generation in the development and renaissance of society, bridging the generational gap, increasing the interdependence between different classes of society, contributing to solving societal problems, eliminating deviant and criminal manifestations in society, eliminating the misalignment of values among new generations, spreading good human qualities among children, and ensuring the protection of society from any extremist ideas that harm the security and stability of the country [118].

## 3 Study Methodology

### 3.1 Research design

This study is a descriptive-analytical research [119] that aims to describe and analyze the variables [120] associated with the family's role in instilling social values that enhance children's awareness of the giving. Additionally, the study aims to determine the difficulties that children face in adhering to encouraging social values of volunteering by monitoring, analyzing, and interpreting the data obtained from the study sample. A social survey method was used to collect data and information from children aged 6 to 14, with the aim of obtaining accurate results that reflect the reality of the study variables.

### 3.2 Participants

The research population includes all children aged 6–14 years old in three international schools in the Emirate of Ajman, United Arab Emirates, with an estimated number of 3936 based on data from the Student Affairs Department in the schools where the study was conducted. The choice of these three international schools was based on several logical justifications. Firstly, the administrations of these three schools approved the study to be conducted within their institutions. Secondly, the work team in these three schools was willing to provide all necessary support to complete the study. Thirdly, the diverse student population in these three schools, consisting of children from different nationalities, allowed for the appointment of a representative sample of participants, ensuring that the study's results could be generalized in the UAE and abroad. Finally, informed consent was obtained from the parents of the participating children in these three schools before the study was conducted.

The size of the random sample representing the study community was calculated according to Cochran's Equation, as shown in Equation 1 [121]:

$$n=\frac{n_{o}}{1+\frac{\left(n_{o}-1\right)}{N}}$$

The sample size in restricted populations, which refers to the research population, is denoted by n, whereas the sample size in infinite or open communities is marked by n_0_, N denotes the size of the research population, which identified 3936 children by official data from the Student Affairs Department in three international schools in the United Arab Emirates. The researcher used Smith's Equation to determine n_0_, as shown in Equation 2 [122,123].

$$n_{o}=\frac{Z^{2}\sigma^{2}}{e^{2}}$$

According to, n_0_ is the sample size, z is the coordinate of the standard normal distribution that cuts off an area α at the tails, and the researcher determined α to be 0.99 at the significance level of 1%, which corresponds to a z-value of ±2.58. e is the desired level of precision, which the researcher determined to be one degree. σ is the variance of a trait in the population.

The researcher did the calculations it as following: n_0_ = $\frac{{\;\left(2.58\right)}^{2}\times {\;\left(5.23\right)\;}^{2}}{{\left(1\right)}^{2}\;}$ = 182

And the researcher used the following formula to measure the sample size in the study population:

$$n=\frac{n_{o}}{1+\frac{\;\left(n_{o}\;–\;1\right)}{N\;}}=\frac{182}{\;1+\frac{\left(182\;–\;1\right)}{3936}}=174\;\text{children}$$

So, the researcher applied the sample social survey approach to 174 children.

### 3.3 Study instrument

Questionnaire Tool: The researcher designed a questionnaire form to identify the family's role in instilling social values that enhance children's awareness of the culture of giving while clarifying the difficulties they face. The questionnaire's phrases reflected the objectives and questions of the study. This questionnaire is an original version designed by the researcher based on their scientific readings in the theoretical literature related to the subject of the study. The original version of the questionnaire was in both Arabic and English due to the diversity of nationalities of children residing in the UAE. A three-dimensional Likert scale was used to make it easier for children to answer the questionnaire. The 3-point Likert scale provides freedom for respondents in the quality of the “feedback” observations they provide, where the question is asked to find out the participants' opinion on a particular topic or the degree of respondents' agreement with a particular statement. The answer is usually from a 3-point Likert scale ("agree” and “disagree” as polarity points), in addition to a “neutral” choice. Each opinion is given its weight (weights). This scale is suitable for the type and size of the sample and the nature of the items and sentences included in the questionnaire to gain insight into the feelings, opinions, impressions, and behaviors of the respondents about a set of items that surround the phenomenon or problematic situation being investigated [124,125].

- The validity and reliability of the questionnaire were assessed through the following steps:

Questionnaire validity: The questionnaire tool was presented to a group of 14 specialized scientific arbitrators, who were faculty members at Ajman, Sharjah, and Dubai universities and held doctorates in social work, sociology, and education. The arbitrators were asked to provide their opinions on various aspects of the tool, including its wording, format, and suitability to the topic being measured. The researcher made amendments to the questionnaire based on the arbitrators' suggestions, with phrases that received less than 85% agreement being excluded, and phrases that received more than 75% agreement being added [126,127].

The final version of the questionnaire included 45 phrases organized into two axes, with the first axis consisting of 30 phrases related to the family's role in instilling social values that encourage volunteering in children, and the second axis consisting of 15 phrases related to the difficulties that children face in adhering to social values that encourage their families to volunteer.

Questionnaire reliability was assessed to ensure that if the questionnaire were used or returned under appropriate conditions, it would yield consistent results. The researcher used the test-retest method to verify the reliability of the questionnaire. A small, random sample of 32 children was selected, and the questionnaire was administered to them. After 15 days, the same sample of children was asked to complete the questionnaire again, because all participants remained in the study. The Spearman correlation coefficient was then calculated between the two administrations of the questionnaire. The reliability coefficient was calculated according to Spearman's law of correlation coefficient, as shown in Equation 3 [128]:

$$\text{RS = 1-}\frac{6\;\left(\sum D2\right)}{n\;\left(n2-1\right)}$$

Table 1 presents the correlation coefficients of the questionnaire and its axes, which were found to be high, indicating the stability of the questionnaire. The self-validity factor was calculated by taking the square root of the stability factor of the questionnaire. The self-validity of the questionnaire was found to be 79.3, equivalent to 89.1%. Therefore, the questionnaire tool is characterized by stability. Thus, all items of the questionnaire tool's axes have a high degree of validity and reliability.

- Merits and Demerits of the Questionnaire Tool

**Table 1:** Questionnaire stability and its variables.

Questionnaire Variables	Reliability (Rs)	Validity (Vs)
Family's role in instilling social values that enhance children's awareness of the culture of giving.	0.755	0.869
Difficulties that children face in adhering to the encouraging social values of volunteering by their families.	0.831	0.912
**TOTAL**	0.793	0.891

The questionnaire tool used in this study has merits and demerits. One of the main advantages of the questionnaire tool is that it is a cost-effective and efficient method of collecting data from a large sample of participants. Additionally, the use of a Likert scale in the questionnaire tool allows for standardized responses to be collected, which can be easily analyzed using statistical methods [129,130].

However, there are also some limitations to the questionnaire tool. One of the potential demerits is that the responses obtained may be influenced by social desirability bias, where some participants may provide answers that they think are socially acceptable. Additionally, the use of closed-ended questions in the questionnaire tool may limit the depth of information obtained from participants [131,132].

Despite these limitations, the questionnaire tool employed in this study was meticulously crafted and validated to ensure its reliability and validity. The validity of the questionnaire tool was assessed by presenting it to a group of expert scientific arbitrators who scrutinized its wording, format, and appropriateness. The reliability of the questionnaire tool was confirmed through the test-retest method, revealing high correlation coefficients for the questionnaire and its dimensions [133,134]. Ultimately, the meticulous design and validation of the questionnaire tool employed in this study guarantee its reliability and validity in collecting data on the role of the family in fostering social values and cultivating a culture of giving back among children.

### 3.4 Data collection measures and Statistical analysis

Data was collected through the questionnaire from 174 children in the age group of 6–14 years old in elementary and middle stages. The data collection period spanned 11 months, from May 2021 to April 2022, to determine children's views on the nature of their family's role in instilling social values that enhance their awareness of the culture of giving, as well as to identify the difficulties that children face in adhering to social values that encourage volunteering. Additionally, the study aimed to determine the extent to which children's awareness of social values that establish the culture of giving differs according to gender, age, educational stage, and cultural level of the family. The researcher used a three-dimensional Likert scale for each statement, with the following response options.

o If the respondent's answer to the phrase " Agree " gets 3 marks
o If the respondent's answer to the phrase " Neutral " gets 2 marks
o If the respondent's answer to the phrase " Disagree” gets 1 mark.

and that determined the degree to the participants responded to each statement of the questionnaire.

And since the questionnaire consisted of 45 statements on two axes, and the number of the study sample members are 174 children, the following is determined:

First, the researcher calculated the level of the family role axis in the questionnaire, which consisted of 30 sentences, the range will be calculated by determining the difference between the highest and lowest value that can be obtained as follows:

Highest value: 174 x 30 x 3 = 15660.

Minimum value: 174 x 30 x 1 = 5220.

So the range = 15660 - 5220 = 10440.

Thus, 10440/3 = 3480.

Accordingly, the levels of the family role axis will be divided as follows.

- Low axis level (5220–8700)
- Middle axis level (8701–12180)
- High axis level (12181–15660)

Second, as for the difficulties axis in the questionnaire, which consisted of 15 sentences, the range will be calculated by determining the difference between the highest and lowest value that can be obtained as follows.

#### 3.4.1 Highest value: 174 x 15 x 3 = 7830

Minimum value: 174 x 15 x 1 = 2610.

So the range = 7830 - 2610 = 5220.

Thus, 5220/3 = 1740.

Accordingly, the levels of the difficulties axis will be divided as follows.

- Low axis level (2610–4350)
- Middle axis level (4351–6090)
- High axis level (6091–7830)

And to analyze the data and its interpretation, the researcher used the analytical tools of the SPSS program. Besides, some statistical coefficients to address the current study questions, which were (mean, frequencies, percentages, total weights). In addition, the researcher's used a one-way ANOVA test to determine the importance of variations in averages.

### 3.5 Informed consent

Before starting the survey, all Children's parents gave their informed agreement to have the questionnaire filled out by their children. The data of the participants were thoroughly examined without exposing their identities.

## 4 Study results

### 4.1 Demographic characteristics of the participating children

Table 2 presents the demographic characteristics of the children who participated in the study conducted in three international schools in the Emirate of Ajman, United Arab Emirates. The majority of the children in the study population were girls, followed by boys. Moreover, most of the children were aged between 12 and 14 years, followed by those aged between 9 and 11 years, and then those aged between 6 and 8 years. The results also showed that the largest percentage of children were in the middle stage, followed by the elementary stage. Regarding the parents' educational level, the highest percentage of parents had a high qualification, followed by intermediate qualification, and then master's or Ph.D. degree. Conversely, the smallest percentage of parents had no education. Additionally, the results showed that the largest percentage of families had a normal cultural level, followed by high cultural level, and then poor cultural level.

**Table 2:** Demographic characteristics of the participating children (N = 174).

Variables	Statement	Frequencies	Percentage %
**Gender**	Boy	72	41.4%
	Girl	102	58.6%
**Age**	6–8	36	20.7%
	9–11	41	23.6%
	12–14	97	55.7%
**Educational stage**	Elementary stage	83	47.7%
	Middle stage	91	52.3%
**Parents' educational level**	Illiterate	11	6.3%
	Intermediate qualification	52	29.9%
	High qualification	85	48.9%
	Master's or Ph.D.	26	14.9%
**The family cultural level**	High	30	17.3%
	Normal	121	69.5%
	Poor	23	13.2%

Note: The researcher divided the ages of the children participating in the study, as shown in the table, into three categories to explore the extent of children's awareness, which coincides with their intellectual development year after year, about the meaning of social values associated with volunteering.

### 4.2 The Family's role in instilling social values that enhance Children's awareness of the culture of giving

Table 3 shows that, according to the children's own perspectives, the family's role in instilling social values that enhance children's awareness of the culture of giving was at a moderate level, with a total weight of 9705 and a weighted relative weight of 61.97%. Analysis of the responses of children participating in this study revealed that the family, especially parents, helped children learn many positive human values and qualities. Perhaps the most important of these values was that one of God's greatest tests for humankind is the extent of their endurance in the face of affliction and faith in God's decree and destiny. The family also taught children that charity and caring for the poor are principles that all heavenly religions urge to apply societally. Additionally, the values and culture of their society instilled in them a commitment to decent moral behavior with members of their society in general and the needy in particular. The family also taught children that meeting the needs of others and restoring rights to their owners is a patriotic duty toward their community. Furthermore, the family taught children that listening to the experiences of their elderly ancestors increases their life experiences and strengthens the mutual respect between their generation and the elderly generation. The family also instilled in children the belief that all human beings are equal regardless of religion, gender, or color, and that they should respect construction workers, cleaners, and other simple business owners. Additionally, the family taught children that love and belonging to the homeland are measured by the strength of social solidarity among its inhabitants through the help of the rich to the poor. Children were also taught to seek to participate in awareness campaigns and blood donation campaigns, and to strive to correct their mistakes, especially with those in need, even if some criticize them. The family also taught children to take into account the words and terms they use when communicating with disadvantaged groups in their society, and to seek to strengthen their relationship with their relatives, even if they are from low-income backgrounds. Children were also taught that the family is the first person responsible for shaping the charitable and humanitarian aspect of their souls, and that seeking to know the requirements of poor families in their community to meet their needs and provide services to them is a national duty.

**Table 3:** The family's role in instilling social values that enhance children's awareness of the culture of giving (N = 174).

Family's role in instilling social values that enhance children's awareness of the culture of giving	Response						Total weights	Weighted relative weight %	percentage %	Ranking
	Agree		Neutral		Disagree					
	*f*	%	*f*	%	*f*	%				
**- “My family taught me to believe in the equality of human beings regardless of religion, gender, or color."**	124	71.3	30	17.2	20	11.5	452	86.6	4.66	6
**- “My family taught me that listening to the experiences of our elderly ancestors increases my life experiences and strengthens mutual respect between our generation and theirs."**	131	75.3	25	14.4	18	10.3	461	88.3	4.75	4
**- “My family taught me that when communicating with disadvantaged groups in my society, I should take into account the words and terms I use."**	43	24.7	45	25.9	86	49.4	305	58.4	3.14	12
**- “My family taught me that the values and culture of our society instill in us a commitment to decent moral behavior with members of our society in general and the needy in particular."**	131	75.3	38	21.8	5	2.9	474	90.8	4.88	3
**- “My family taught me that seeking to know the requirements of poor families in my community to meet their needs and provide services to them is a national duty."**	33	19	46	26.4	95	54.6	286	54.8	2.95	15
**- “My family taught me to be cautious when dealing with the homeless and beggars, as some may falsely claim need."**	97	55.7	39	22.4	38	21.8	407	78	4.19	8
**- “My family taught me that one of God's greatest tests for humankind is the extent of our endurance in the face of affliction and faith in God's decree and destiny."**	170	97.7	4	2.3	0	0	518	99.2	5.34	1
**- “My family taught me to respect construction workers, cleaners, and other individuals in simple business occupations."**	105	60.3	48	27.6	21	12.1	432	82.8	4.45	7
**- “My family taught me that charity and caring for the poor is a principle that all heavenly religions urge to apply societally."**	147	84.5	27	15.5	0	0	495	94.8	5.10	2
**- “My family taught me that love and belonging to the homeland are measured by the strength of social solidarity among its inhabitants through the help of the rich to the poor."**	88	50.6	41	23.6	45	25.8	391	74.9	4.02	9
**- “My family taught me that the family is the first person responsible for shaping the charitable and humanitarian aspect in our souls."**	53	30.5	12	6.9	109	62.6	292	55.9	3.01	14
**- “My family taught me to strive to correct my mistakes, especially with those in need, even if some criticize me."**	16	9.2	106	60.9	52	29.9	312	59.7	3.21	11
**- “My family taught me that meeting the needs of others and restoring their rights is a patriotic duty toward my community."**	128	73.6	31	17.8	15	8.6	461	88.3	4.75	4
**- “My family taught me to seek to strengthen my relationship with my relatives, even if they are from low-income backgrounds."**	46	26.4	34	19.6	94	54	300	57.5	3.09	13
**- “My family taught me to seek to volunteer in NGOs and donate to them within the limits of my capabilities."**	17	9.8	54	31	103	59.2	262	50.2	2.70	20
**- “My family taught me to seek to participate in awareness campaigns and blood donation campaigns."**	62	35.6	49	28.2	63	36.2	347	66.5	3.58	10
**- “My family taught me the necessity of taking care of orphans, the elderly, and other groups most in need and affected by my community."**	24	13.8	61	35.1	89	51.1	283	54.2	2.92	16
**- “My family taught me to give alms at the times that God has commanded me."**	27	15.5	48	27.6	99	56.9	276	52.9	2.84	17
**- “My family taught me to make time for volunteering, despite a busy schedule."**	21	12.1	39	22.4	114	65.5	255	48.9	2.63	22
**- “My family taught me to believe that money is a resource that should be used wisely, and that saving money is a responsible action."**	20	11.5	52	29.9	102	58.6	266	51	2.74	19
**- “My family taught me to believe that the care and attention that parents provide to their children will have positive results in the future, such as honoring and caring for children to their parents."**	15	8.6	43	24.7	116	66.7	247	47.3	2.55	24
**- “My family taught me to consider it my duty to volunteer in rescue and relief campaigns for the afflicted in neighboring countries as a means of participating in the protection of human rights globally."**	13	7.5	23	13.2	138	79.3	223	42.7	2.30	29
**- “My family taught me to be interested in discussing the most important social and humanitarian issues in my country with those close to my family."**	13	7.5	36	20.7	125	71.8	236	45.2	2.43	27
**- “My family taught me to always strive to overcome restrictions imposed by traditions of our society, such as respecting the rights of the weak and meeting their needs."**	2	1.2	42	24.1	130	74.7	220	42.1	2.27	30
**- “My family taught me to donate material and in-kind items to charities, in cooperation with my family and close friends."**	18	10.3	65	37.4	91	52.3	275	52.7	2.83	18
**- “My family taught me that it is my duty to take a serious stand against negative behaviors towards the poor."**	18	10.4	27	15.5	129	74.1	237	45.4	2.44	26
**- “My family taught me to have an interest in reading the constitution of my country to learn about the forms of care guaranteed to weak and needy groups in my society."**	19	10.9	41	23.6	114	65.5	253	48.5	2.61	23
**- “My family did teach me to believe that a productive time is solely measured by the amount of money made."**	9	5.2	44	25.3	121	69.5	236	45.2	2.43	27
**- “My family taught me that social media and media programs have a weak interest in providing distinguished competencies in human services."**	16	9.2	38	21.8	120	69	244	46.7	2.52	25
**- “My family taught me that the curricula I study at school do not fully develop the voluntary aspect of my personality."**	17	9.8	51	29.3	106	60.9	259	49.6	2.67	21
Total							**9705**		**100%**	
Weighted relative weight of the variable	**61.97 %**									
Level of weight representation	**Moderate**									

Note: The total weights are the sum of the multiplication product (the number of frequencies for each category × the score of each category on the Likert scale) for each statement. The weighted relative weight is the result of dividing the sum of the weights for each phrase ÷ the maximum sum of the weights of one phrase. The percentage is the result of dividing the sum of the weights for each phrase ÷ the sum of the weights for all the axis phrases.

### 4.3 The difficulties that children face in adhering to the encouraging social values of volunteering by their families

According to Table 4, the difficulties that children face in adhering to the encouraging social values of volunteering by their families are at a moderate level, with a total weight of 4822 and a weighted relative weight of 61.58%. Analysis of the responses of children participating in this study revealed that the most challenging aspect of adhering to social values that encourage giving by their families is family disputes between the child's parents, which do not allow the child to participate in human services. Furthermore, some families do not explain the nature and mechanism of applying encouraging social values of giving to their children, while others lack interest in practicing human services, leading to a generation of children who do not appreciate the value of giving. In addition, some families do not clarify the procedures for their children to join human services, while others believe that human services negatively affects their children's studies. Moreover, some families do not help their children understand the conditions and criteria for selection by their school to participate in service activities. Similarly, some families do not encourage their children to follow media programs that host distinguished children in the field of human services, and some families refuse their children's interest in knowing about voluntary programs. Some families teach their children that the customs and traditions of their society do not support participation in protecting vulnerable groups, as that is the responsibility of the state, not the individual. Additionally, some families refuse to attend their children to seminars, training courses, and workshops implemented by their schools regarding encouraging volunteerism. Finally, some families teach their children to prioritize their personal interests over the public interest, which is based on serving people they do not know.

**Table 4:** The difficulties that children face in adhering to the encouraging social values of volunteering by their families (N = 174).

Difficulties that children face in adhering to the encouraging social values of volunteering	Response						Total weights	Weighted relative weight %	percentage %	Ranking
	Agree		Neutral		Disagree					
	*f*	%	*f*	%	*f*	%				
**- “My parents did not explain the procedures to follow in order to participate in human services."**	64	36.8	49	28.2	61	35	351	67.2	7.28	4
**- “Family disputes between my parents prevent me from being able to participate in human services."**	84	48.3	61	35.1	29	16.6	403	77.2	8.36	1
**- “I feel frustrated because my family does not appreciate my efforts in volunteering."**	8	4.6	31	17.8	135	77.6	221	42.3	4.58	15
**- “My parents lack the interest to practice in actual human services, and as a result, I am like them."**	97	55.7	4	2.3	73	42	372	71.3	7.71	3
**- “My family does not encourage me to follow media programs that feature outstanding children in the field of volunteering."**	40	23	81	46.6	53	30.4	335	64.2	6.95	7
**- “My family taught me that volunteering is a waste of time and negatively affects my studies."**	27	15.5	115	66.1	32	18.4	343	65.7	7.11	5
**- “My family refuses to attend seminars, training courses, and workshops that my school implements to encourage volunteerism."**	25	14.4	91	52.3	58	33.3	315	60.3	6.53	10
**- “My family has not convinced me that volunteering can positively develop my personality, so I do not practice it."**	21	12.1	60	34.5	93	53.4	276	52.9	5.72	13
**- “My family rejects my interest in learning about volunteer programs in order to participate in them."**	11	6.3	139	79.9	24	13.8	335	64.2	6.95	7
**- “My family taught me that the customs and traditions of my society do not support participation in protecting vulnerable groups, as that is the responsibility of the state and not the individual."**	38	21.8	76	43.7	60	34.5	326	62.5	6.76	9
**- “The lack of commitment by some members of my family with the etiquette of dealing with the needy reduces my desire to volunteer."**	13	7.5	52	29.9	109	62.6	252	48.3	5.23	14
**- “My family taught me to prioritize my personal interest in serving myself over the common good, which is based on serving people I do not know."**	19	10.9	68	39.1	87	50	280	53.6	5.81	11
**- “My family does not help me understand the terms and criteria for selecting students to participate in service activities implemented by my school administration."**	45	25.9	72	41.4	57	32.7	336	64.4	6.97	6
**- “My family did not explain to me the nature and mechanism of applying encouraging social values of volunteering."**	102	58.6	22	12.6	50	28.8	400	76.6	8.30	2
**- “My family does not allow me to donate to the poor."**	28	16.1	45	25.9	101	58	277	53.1	5.74	12
Total							**4822**		**100%**	
Weighted relative weight of the variable	**61.58%**									
Level of weight representation	**Moderate**									

Note: The total weights are the sum of the multiplication product (the number of frequencies for each category × the score of each category on the Likert scale) for each statement. The weighted relative weight is the result of dividing the sum of the weights for each phrase ÷ the maximum sum of the weights of one phrase. The percentage is the result of dividing the sum of the weights for each phrase ÷ the sum of the weights for all the axis phrases.

### 4.4 Variations in Children's awareness of social values encouraging giving

To analyze the differences between the averages of the degree of children's awareness of the social values that entrench their culture of giving differ according to gender, age, educational stage, parents' educational level, and family cultural level, the researcher used an independent one-way ANOVA test.

Table 5 showed there are statistically significant differences in the degree of children's awareness of the social values that entrench their culture of giving according to the variable of gender, where **F**_**Statistic**_ 4.76 is greater than **F**_**Critical**_ 3.84 at the statistical significance level of 0.05. In addition, there are statistically significant differences in the degree of children's awareness of the social values that entrench their culture of giving according to the variable of age, where **F**_**Statistic**_ 3.95 is greater than **F**_**Critical**_ 3.00 at the statistical significance level of 0.05. Besides, there are statistically significant differences in the degree of children's awareness of the social values that entrench their culture of giving according to the variable of educational qualification, where **F**_**Statistic**_ 6.60 is greater than **F**_**Critical**_ 3.84 at the statistical significance level of 0.05. Also, there are statistically significant differences in the degree of children's awareness of the social values that entrench their culture of giving according to the variable of parents' educational level, where **F**_**Statistic**_ 4.08 is greater than **F**_**Critical**_ 2.60 at the statistical significance level of 0.05. In addition, there are statistically significant differences in the degree of children's awareness of the social values that entrench their culture of giving according to the variable of family cultural level, where **F**_**Statistic**_ 5.46 is greater than **F**_**Critical**_ 3.00 at the statistical significance level of 0.05.

**Table 5:** One-way ANOVA test of children responses.

		Sum of Squares	df	Mean Square	F _Statistic_	F _critical_	Sig. level
**Gender**	Between Groups	147	1	147	4.76	3.84*	Significant
	Within Groups	4586	172	26.66			
	**Total**	4733	173				
**Age**	Between Groups	209	2	104.50	3.95	3.00*	Significant
	Within Groups	4524	171	26.46			
	**Total**	4733	173				
**Educational Qualification**	Between Groups	175	1	175	6.60	3.84*	Significant
	Within Groups	4558	172	26.5			
	**Total**	4733	173				
**Parents' educational level**	Between Groups	318	3	106	4.08	2.60*	Significant
	Within Groups	4415	170	25.97			
	**Total**	4733	173				
**Family cultural level**	Between Groups	284	2	142	5.46	3.00*	Significant
	Within Groups	4449	171	26.02			
	**Total**	4733	173				

Statistically significant at (α 0.05).

## 5 Discussion

The current study aimed to determine the family's role in instilling social values that enhance children's awareness of the culture of giving, and to assess the strength and effectiveness of this role. The study obtained important results that accurately answered the research questions.

Table 3 showed that the family's role in instilling social values that enhance children's awareness of the culture of giving, from the perspective of the children themselves, is at a moderate level. Findings highlighted the importance of the family's role in consolidating values such as love, helping others, social solidarity, mutual respect, and cooperation in order to extend a helping hand to those in need. This is consistent with a study by Handayani 2021, which showed that the family is the cornerstone of society and is primarily responsible for the process of socialization [135]. Moreover, Suri's 2021 study demonstrated that the process of socialization carried out by parents should include introducing children to positive social values and their significance within society [136]. Additionally, Salmiati and Zaman's 2021 study emphasized that parents should support their children in engaging in human services [137]. However, studies conducted by Grönlund 2011 and Wood 2017 have indicated that children's motivation to engage in human services is not solely dependent on their parents' encouragement but can also be influenced by their own intrinsic motivation and personal interests [138,139].

It is worth noting that, despite the importance of the family's role in instilling social values that enhance children's awareness of the culture of giving, the current study found that the degree of this role was moderate. This suggests that families need to provide more attention and effort to instill authentic human qualities in the hearts of their children. These qualities can help develop their personalities and encourage them to embrace values such as kindness and volunteering time, effort, and money to extend a helping hand to vulnerable groups in society. This finding is consistent with studies by Brata 2021 and Rubini 2021, which demonstrated that constructive social values, such as cooperation, equality, sincerity, patience, and justice, have a significant role in enhancing individuals' personalities and making them effective members of society who can benefit both themselves and others [140,141].

According to Tables 4 and it has been observed that children face moderate difficulties in adhering to social values of volunteering. Findings suggested that family disputes are a prominent hindrance for children in adopting social values that encourage volunteering within their families, due to the unfavorable living conditions imposed by their parents. This result is consistent with a previous study by McKinley 2022, which also highlighted various difficulties that children face within their families, including parental disputes [142].

Moreover, some families fail to explain the nature and mechanism of encouraging social values related to human services to their children. In addition, families may not support their children's engagement in human services, viewing it as a hindrance to their academic careers. This finding is consistent with a study by Al-Hassan 2021 which highlighted the negative impact that lack of support and encouragement can have on children's values and behaviors. This underscores the importance of directing families towards educational methods that emphasize effective communication between parents and children, with parents serving as models and high ideals for their children's moral, behavioral, and voluntary behavior [143].

Furthermore, Table 5 showed there are statistically significant differences in children's awareness of social values that promote giving based on gender, age, educational qualifications, parental education level, and family cultural level at a significance level of 0.05. These findings are consistent with study by Kress 2018, which suggest that an individual's acquisition of social values can vary based on their educational level, age, and gender [144]. However, studies by Hofstede 2011 and Gifford & Nilsson 2014 and indicate that an individual's acquisition of social values does not vary based on their gender and age, but rather on their cultural level [145,146].

Despite the valuable insights gained from this study, it is important to acknowledge the limitations of the research.

⁃ Location limitation: The study was conducted in international schools in the Emirate of Ajman, UAE.
⁃ Time limitation: The study was conducted from May 2021 to April 2022.
⁃ Human limitation: The study was limited to a representative sample of 174 children from international schools in the Emirate of Ajman.
⁃ Literary limitations: There is a scarcity of previous research studies on the topic of this study in the Arab world, especially.
⁃ Study focus: The study aimed to determine the family's role in instilling social values that enhance children's awareness of the culture of giving and to assess the strength and effectiveness of this role.

In light of these limitations, suggestions for future research are as follows:

Future research should aim to explore the influence of external factors such as school or community environments on children's awareness of social values promoting giving. This can be achieved by conducting studies in different locations with larger and more diverse samples, utilizing multiple methods of data collection, and incorporating other contextual factors. These approaches will provide a more comprehensive understanding of the topic.

## 6 Conclusion

Based on the findings of the study, the following conclusions can be drawn.

⁃ The family plays a crucial role in instilling social values that enhance children's awareness of Culture of giving. However, the family's role in this area is currently rated as moderate, indicating that there is room for improvement.
⁃ Children face difficulties in adhering to the social values that encourage volunteering, which can be attributed to factors such as family disputes and a lack of interest in explaining the nature and mechanism of applying these values.
⁃ Statistically significant differences were found in children's awareness of social values based on gender, age, educational stage, parents' educational level, and family cultural level. This highlights the need for targeted interventions that take into account these demographic factors.

In light of these conclusions, the following recommendations are suggested.

• Activating the role of families and educational institutions in organizing field visits for children to participate in voluntary and humanitarian programs can be an effective way to deepen children's understanding of vulnerable groups in society and the importance of providing support and assistance to them.
• Increasing the family's role in instilling social values that enhance children's awareness of the culture of giving, through holding seminars and meetings that support family communication and interaction.
• Encouraging families to participate in field visits organized by educational institutions that promote a culture of giving, and to interact constructively with vulnerable groups in society.
• Raising awareness among parents of their duties towards their children and the importance of following up on their children's activities, including their social media use and the people they interact with. Because bad friends in a child's life may affect him negatively by giving him ideas and convictions that contradict religion and societal morals
• Educating families about the need to pay attention to teaching the values of love, compassion, cooperation, and teamwork in the hearts of their children, and encouraging families to train their children to respect and appreciate every human being.
• Activating the role of educational institutions in promoting volunteering, and providing targeted interventions that take into account demographic factors such as gender, age, educational stage, parents' educational level, and family cultural level.

By implementing these recommendations, families and educational institutions can work together to instill positive social values in children and build a compassionate and committed future generation capable of giving back to society.

## Funding statement

This research did not receive any specific grant from funding agencies in the public, commercial, or not-for-profit sectors.

## Additional information

No additional information is available for this paper.

## Data availability statement

Data will be made available on request.

## CRediT authorship contribution statement

**Walaa Elsayed:** Writing – review & editing, Writing – original draft, Visualization, Validation, Supervision, Software, Resources, Project administration, Methodology, Investigation, Funding acquisition, Formal analysis, Data curation, Conceptualization.

## Declaration of competing interest

The authors declare that they have no known competing financial interests or personal relationships that could have appeared to influence the work reported in this paper.

## References

[1] Fenton J. (2016).

[2] Elsayed W. (2021). The negative effects of social media on the social identity of adolescents from the perspective of social work. Heliyon.

[3] Wyness M. (2019).

[4] Elsayed W. (2020). Students and the risk of virtual relationships in social media: improving learning environments. International Journal of Emerging Technologies in Learning (iJET).

[5] Abbas E.W., Mutiani M., Nugraha D.S. (2018). The 3rd ISSSHE International Seminar Sosial Studies & History Education" Promoting the 21st Century Skills in Social Studies Learning.

[6] Elsayed W. (2022). An analytical view from the perspective of method community organization of the reality of women's volunteer work in the emirate of ajman in the UAE. Dirasat Hum. Soc. Sci..

[7] Ikhwan A., Biantoro O.F., Rohmad A. (2019). The role of the family in internalizing islamic values. Dinamika Ilmu.

[8] Pérez-Fuentes M.D.C., Molero Jurado M.D.M., Barragán Martín A.B., Gazquez Linares J.J. (2019). Family functioning, emotional intelligence, and values: analysis of the relationship with aggressive behavior in adolescents. Int. J. Environ. Res. Publ. Health.

[9] Twito L., Knafo-Noam A. (2020). Beyond culture and the family: evidence from twin studies on the genetic and environmental contribution to values. Neurosci. Biobehav. Rev..

[10] Barbera F., Shi H.X., Agarwal A., Edwards M. (2020). The family that prays together stays together: toward a process model of religious value transmission in family firms. J. Bus. Ethics.

[11] Elsayed W. (2021). Covid-19 pandemic and its impact on increasing the risks of children's addiction to electronic games from a social work perspective. Heliyon.

[12] Vogl T.S., Freese J. (2020). Proceedings of the National Academy of Sciences.

[13] Anggadwita G., Profityo W.B., Alamanda D.T., Permatasari A. (2019). Cultural values and their implications to family business succession: a case study of small Chinese-owned family businesses in Bandung, Indonesia. J. Fam. Bus. Manag..

[14] Kenter J.O., O'Brien L., Hockley N., Ravenscroft N., Fazey I., Irvine K.N., Williams S. (2015). What are shared and social values of ecosystems?. Ecol. Econ..

[15] Ives C.D., Kidwell J. (2019). Religion and social values for sustainability. Sustain. Sci..

[16] Rasoolimanesh S.M., Iranmanesh M., Amin M., Hussain K., Jaafar M., Ataeishad H. (2020). Are functional, emotional and social values interrelated? A study of traditional guesthouses in Iran. Int. J. Contemp. Hospit. Manag..

[17] Lake J., Gerrans P., Sneddon J., Attwell K., Botterill L.C., Lee J.A. (2021). We're all in this together, but for different reasons: social values and social actions that affect COVID-19 preventative behaviors. Pers. Indiv. Differ..

[18] Torbica A., Fornaro G., Tarricone R., Drummond M.F. (2020). Do social values and institutional context shape the use of economic evaluation in reimbursement decisions? An empirical analysis. Value Health.

[19] de Vries W.T., Voß W., Raumforschung und Raumordnung (2018).

[20] Boone C., Buyl T., Declerck C.H., Sajko M. (2020). A neuroscience-based model of why and when CEO social values affect investments in corporate social responsibility. Leader. Q..

[21] Litina A., Palivos T. (2016). Corruption, tax evasion and social values. J. Econ. Behav. Organ..

[22] Huggins R., Thompson P. (2015). Local entrepreneurial resilience and culture: the role of social values in fostering economic recovery. Camb. J. Reg. Econ. Soc..

[23] Rahayu N., Warto W., Sudardi B., Wijaya M. (2020). Dynamics of social values and teaching in the global era: beyond sekaten tradition in surakarta kingdom. J. Soc. Stud. Educ. Res..

[24] Kronenberg J., Andersson E. (2019). Integrating social values with other value dimensions: parallel use vs. combination vs. full integration. Sustain. Sci..

[25] Arikan G., Ben‐Nun Bloom P. (2015). Social values and cross‐national differences in attitudes towards welfare. Polit. Stud..

[26] Agyabeng-Mensah Y., Ahenkorah E., Afum E., Dacosta E., Tian Z. (2020). Green warehousing, logistics optimization, social values and ethics and economic performance: the role of supply chain sustainability. Int. J. Logist. Manag..

[27] Nahuelhual L., Ochoa F.B., Rojas F., Díaz G.I., Carmona A. (2016). Mapping social values of ecosystem services: what is behind the map?. Ecol. Soc..

[28] Eriksson M., van Riper C.J., Leitschuh B., Bentley Brymer A., Rawluk A., Raymond C.M., Kenter J.O. (2019). Social learning as a link between the individual and the collective: evaluating deliberation on social values. Sustain. Sci..

[29] Morris R.L., Deavin G., Hemelryk Donald S., Coleman R.A. (2016). Eco‐engineering in urbanised coastal systems: consideration of social values. Ecol. Manag. Restor..

[30] Gould R.K., Pai M., Muraca B., Chan K. (2019). He ʻike ʻana ia i ka pono (it is a recognizing of the right thing): how one indigenous worldview informs relational values and social values. Sustain. Sci..

[31] Bjärstig T., Kvastegård E. (2016). Forest social values in a Swedish rural context: the private forest owners' perspective. For. Pol. Econ..

[32] Cannas R., Argiolas G., Cabiddu F. (2019). Fostering corporate sustainability in tourism management through social values within collective value co-creation processes. J. Sustain. Tourism.

[33] Littlejohns P., Chalkidou K., Culyer A.J., Weale A., Rid A., Kieslich K., Knight S. (2019). National Institute for Health and Care Excellence, social values and healthcare priority setting. J. R. Soc. Med..

[34] Pickering J., Persson Å. (2020). Democratising planetary boundaries: experts, social values and deliberative risk evaluation in Earth system governance. J. Environ. Pol. Plann..

[35] MacDonald P.A., Murray G., Patterson M. (2015). Considering social values in the seafood sector using the Q-method. Mar. Pol..

[36] Brigandt I. (2015). Social values influence the adequacy conditions of scientific theories: beyond inductive risk. Can. J. Philos..

[37] Busch L. (2016). Individual choice and social values: choice in the agrifood sector. J. Consum. Cult..

[38] Kress G. (2018). Language and Control.

[39] Christie I., Gunton R.M., Hejnowicz A.P. (2019). Sustainability and the common good: catholic Social Teaching and ‘Integral Ecology’as contributions to a framework of social values for sustainability transitions. Sustain. Sci..

[40] Tabernero C., Castillo-Mayén R., Luque B., Cuadrado E. (2020). Social values, self-and collective efficacy explaining behaviours in coping with Covid-19: self-interested consumption and physical distancing in the first 10 days of confinement in Spain. PLoS One.

[41] Elsayed W. (2021). Social work practices in the multiethnic urban reality of covid-19 in the Middle East: the case of UAE. Journal of Ethnic and Cultural Studies.

[42] Bal B.S., Brenner L.H. (2015). Medicolegal sidebar: the law and social values: conformity to norms. Clin. Orthop. Relat. Res..

[43] Raymond I.J., Raymond C.M. (2019). Positive psychology perspectives on social values and their application to intentionally delivered sustainability interventions. Sustain. Sci..

[44] Jonason P.K., Koehn M.A., Bulyk R.A., Davis M.D. (2020). Standing out and not fitting in: the Dark Triad traits and social values. J. Soc. Psychol..

[45] Revelli C. (2016). Re-embedding financial stakes within ethical and social values in socially responsible investing (SRI). Res. Int. Bus. Finance.

[46] Village A., Francis L.J. (2016). Measuring the contribution of Roman Catholic secondary schools to students' religious, personal and social values. Journal of Catholic Education.

[47] Silke C., Brady B., Dolan P., Boylan C. (2020). Social values and civic behaviour among youth in Ireland: the influence of social contexts. Ir. J. Sociol..

[48] Younghusband E. (2021).

[49] Zhang W., Yu Y., Wu X., Pereira P., Borja M.E.L. (2020). Integrating preferences and social values for ecosystem services in local ecological management: a framework applied in Xiaojiang Basin Yunnan province, China. Land Use Pol..

[50] Cohen C.J., Chen Y., Orbach H., Freier-Dror Y., Auslander G., Breuer G.S. (2015). Social values as an independent factor affecting end of life medical decision making. Med. Healthc. Philos..

[51] Landry L.N. (2022). The Disability Bioethics Reader.

[52] Ozbey A., Saricam H., Karduz F. (2018). The examination of emotional intelligence, sense of community, perception of social values in gifted and talented students. Journal of Educational Sciences & Psychology.

[53] Cole Z., Holland S., Donohoe H. (2015). A social values typology for comprehensive assessment of coastal zone ecosystem services. Soc. Nat. Resour..

[54] Bjärstig T., Sténs A. (2018). Social values of forests and production of new goods and services: the views of Swedish family forest owners. Small-scale Forestry.

[55] Baba T., Shimada I. (2019). Values and Valuing in Mathematics Education.

[56] Adamek P. (2017). Institut Monumenta Serica.

[57] Резер Т.М. (2021). Social values of students in conditions of digitalization of education and COVID-19. Интеграция образования Integration of Education.

[58] Ofstehage A. (2016). Farming is easy, becoming Brazilian is hard: north American soy farmers' social values of production, work and land in Soylandia. J. Peasant Stud..

[59] Kociszewski K., Graczyk A., Mazurek-Łopacinska K., Sobocińska M. (2020). Social values in stimulating organic production involvement in farming—the case of Poland. Sustainability.

[60] Van den Hoven J., Vermaas P.E., Van de Poel I. (2015). Handbook of Ethics, Values, and Technological Design: Sources, Theory, Values and Application Domains.

[61] Merli R., Preziosi M., Massa I. (2015). Social values and sustainability: a survey on drivers, barriers and benefits of SA8000 certification in Italian firms. Sustainability.

[62] Lin Y.P., Lin W.C., Li H.Y., Wang Y.C., Hsu C.C., Lien W.Y., Petway J.R. (2017). Integrating social values and ecosystem services in systematic conservation planning: a case study in Datuan watershed. Sustainability.

[63] Goldenberg M.J. (2015). Whose social values? Evaluating Canada's ‘death of evidence’controversy. Can. J. Philos..

[64] Rawluk A., Ford R., Anderson N., Williams K. (2019). Exploring multiple dimensions of values and valuing: a conceptual framework for mapping and translating values for social-ecological research and practice. Sustain. Sci..

[65] García-Esparza J.A., Tena P.A. (2020). A GIS-based methodology for the appraisal of historical, architectural, and social values in historic urban cores. Frontiers of Architectural Research.

[66] Leszkowicz E., Linden D.E., Maio G.R., Ihssen N. (2017). Neural evidence of motivational conflict between social values. Soc. Neurosci..

[67] Needham M.D., Szuster B.W., Mora C., Lesar L., Anders E. (2017). Manta ray tourism: interpersonal and social values conflicts, sanctions, and management. J. Sustain. Tourism.

[68] Crafa D., Liu J.Q., Brodeur M.B. (2019). Social values and determinants of cultural fit in Quebec: the roles of ancestry, linguistic group, and mental health status. Front. Psychol..

[69] Reamer F., Reamer F.G. (2018). Social Work Values and Ethics.

[70] Gvelesiani R. (2019).

[71] Terziev V., Arabska E. (2017).

[72] Dodonova V., Dodonov R. (2020). Transformation of social values during a pandemic and problems of global solidarity. Skhid.

[73] Martucci L.A., Fischer-Hübner S., Hartswood M., Jirotka M. (2017). Designing, Developing, and Facilitating Smart Cities.

[74] Widman U., Bjärstig T. (2017). Protecting forests' social values through partnerships. Scand. J. For. Res..

[75] Yung E.H., Chan E.H. (2015). Evaluation of the social values and willingness to pay for conserving built heritage in Hong Kong. Facilities.

[76] Calcagni F., Amorim Maia A.T., Connolly J.J.T., Langemeyer J. (2019). Digital co-construction of relational values: understanding the role of social media for sustainability. Sustain. Sci..

[77] Baghurst B., Lukatelich R., Smith D., Begg G., Lewis R., Smith R. (2017). Findings from the Great Australian Bight Research Program–an integrated study of environmental, economic and social values. The APPEA Journal.

[78] Bogdan S.M., Stupariu I., Andra-Toparceanu A., Năstase I.I. (2019). Mapping social values for cultural ecosystem services in a mountain landscape in the Romanian carpathians. Carpathian Journal of Earth and Environmental Sciences.

[79] Menard R. (2016). Analysing social values in identification; a framework for research on the representation and implementation of values. J. Theor. Soc. Behav..

[80] Sténs A., Bjärstig T., Nordström E.M., Sandström C., Fries C., Johansson J. (2016). In the eye of the stakeholder: the challenges of governing social forest values. Ambio.

[81] Cheung C.H.W., Kennedy K.J., Leung C.H., Hue M.T. (2018). Religious engagement and attitudes to the role of religion in society: their effect on civic and social values in an Asian context. Br. J. Relig. Educ..

[82] Al-Mosa N.A. (2015). Role of social networks in developing religious and social values of the students of the world islamic sciences & education university. Int. Educ. Stud..

[83] Smith M.B. (2017).

[84] Bechmann A. (2017). Proceedings of the 50th Hawaii International Conference on System Sciences.

[85] Brenner J.L., Kabakyenga J., Kyomuhangi T., Wotton K.A., Pim C., Ntaro M., Singhal N. (2011). Can volunteer community health workers decrease child morbidity and mortality in southwestern Uganda? An impact evaluation. PLoS One.

[86] Elsayed W. (2022). The efficiency of social workers in the application of quality standards for the social integration programs for children of Asperger's syndrome in centers of people of determination from perspective of the community organization method. Dirasat Hum. Soc. Sci..

[87] Schmidt A. (2007). Volunteer child soldiers as reality: a development issue for Africa. New school economic review.

[88] Strickland-Munro J., Moore S., Kobryn H., Palmer D. (2015). Kimberley Marine Research Program Node of the Western Australian Marine Science Institution.

[89] Elsayed W. (2023). The impact of campus housing problems faced by female expatriate students on the level of their academic achievement. Int. Soc. Sci. J..

[90] Johansson T., Segerstedt E., Olofsson T., Jakobsson M. (2016). Revealing social values by 3D city visualization in city transformations. Sustainability.

[91] Elsayed W., Konstantin S., Yulia G. (2022). Religious practices in the effectiveness of social service workers: a subjective assessment. Publ. Organ. Rev..

[92] Zahle J. (2018). Values and data collection in social research. Philos. Sci..

[93] Özdenk S., Karabulut E.O. (2018). Examination of youth team athletes' social values according to some variables. Int. J. High. Educ..

[94] Zappavigna M., Martin J.R. (2018). # Communing affiliation: social tagging as a resource for aligning around values in social media. Discourse, context & media.

[95] Sai̇hu, S. S., & Islamy, A. (2019). Exploring the values of social education in the qur’an. Academic Knowledge, 3(1), 59-84.‏].

[96] Mostafavi, H., Arab, M., & Rashidian, A. (2017). Social values in health prioritizing: the analysis of national documents of Iran. Health and Development Journal, 6(3), 191-205.‏].

[97] Suh W.S., Kahle L.R. (2017). The Routledge Companion to Consumer Behavior.

[98] Jang I., Jun M., Lee J.E. (2017). Economic actions or cultural and social decisions? The role of cultural and social values in shaping fertility intention. International Review of Public Administration.

[99] Chatterjee M.B., Baumann N., Koole S.L. (2017). Feeling better when someone is alike: poor emotion regulators profit from pro‐social values and priming for similarities with close others. J. Pers..

[100] Elsayed W. (2023). Behind closed doors: exploring the consequences of parents staying at home during the COVID-19 pandemic on the prevalence of parental violence against children. International and multidisciplinary journal of social sciences.

[101] Uzefovsky F., Döring A.K., Knafo‐Noam A. (2016). Values in middle childhood: social and genetic contributions. Soc. Dev..

[102] Gazzola P., Mella P. (2018). Contemporary Trends and Challenges in Finance.

[103] Erhardt N., Martin-Rios C., Bolton J., Luth M. (2022). Doing well by creating economic value through social values among craft beer breweries: a case study in responsible innovation and growth. Sustainability.

[104] Anastasiu I., Bîgu D. (2018). Social values and cultural diversity: a comparative view. Econ. Manag. Financ. Mark..

[105] Gao Q. (2017). Social values and rock art tourism: an ethnographic study of the Huashan rock art area (China). Conserv. Manag. Archaeol. Sites.

[106] Shi S., Chen Y., Chow W.S. (2016). Key values driving continued interaction on brand pages in social media: an examination across genders. Comput. Hum. Behav..

[107] Strand P.S., Pula K., Downs A. (2015). Social values and preschool behavioral adjustment: a comparative investigation of Latino and European American preschool children. Cult. Divers Ethnic Minor. Psychol..

[108] Grøgaard B., Colman H.L. (2016). Interpretive frames as the organization's “mirror”: from espoused values to social integration in MNEs. Manag. Int. Rev..

[109] Santana S., Morwitz V. (2015).

[110] Miller V.J., Lee H. (2020). Social work values in action during COVID-19. J. Gerontol. Soc. Work.

[111] Han S.H., Chen C.H.S., Lee T.J. (2021). The interaction between individual cultural values and the cognitive and social processes of global restaurant brand equity. Int. J. Hospit. Manag..

[112] Lee K.E., Kendal D. (2017). Urban Biodiversity.

[113] De Silva M., Gokhberg L., Meissner D., Russo M. (2021). Addressing societal challenges through the simultaneous generation of social and business values: a conceptual framework for science-based co-creation. Technovation.

[114] Sanderson J., Frederick E., Stocz M. (2016). When athlete activism clashes with group values: social identity threat management via social media. Mass Commun. Soc..

[115] Marzano M., Ambrose-Oji B., Hall C., Moseley D. (2020). Pests in the city: managing public health risks and social values in response to oak processionary moth (Thaumetopoea processionea) in the United Kingdom. Forests.

[116] Abbott P., Teti A., Sapsford R. (2020). The tide that failed to rise: young people's politics and social values in and after the Arab uprisings. Mediterr. Polit..

[117] Sayer A. (2017). Values within reason. Canadian Review of Sociology/Revue canadienne de sociologie.

[118] Cannas R. (2017). Collaborative Economy and Tourism.

[119] Blunch N.J. (2012).

[120] Flaskerud J.H. (2012). Cultural bias and Likert-type scales revisited. Issues Ment. Health Nurs..

[121] Stehman S.V. (2012). Impact of sample size allocation when using stratified random sampling to estimate accuracy and area of land-cover change. Remote Sensing Letters.

[122] Conrad P.R., Girolami M., Särkkä S., Stuart A., Zygalakis K. (2017). Statistical analysis of differential equations: introducing probability measures on numerical solutions. Stat. Comput..

[123] Hatcher L. (2013).

[124] Joshi A., Kale S., Chandel S., Pal D.K. (2015). Likert scale: explored and explained. Br. J. Appl. Sci. Technol..

[125] Taherdoost H. (2019). What is the best response scale for survey and questionnaire design; review of different lengths of rating scale/attitude scale/Likert scale. Hamed Taherdoost.

[126] De Veaux R.D., Velleman P.F., Bock D.E., Vukov A.M., Wong A.C., Burkett C. (2005).

[127] Meloun M., Militký J. (2011).

[128] Rugg G. (2007).

[129] Rubin A., Babbie E.R. (2016).

[130] Creswell J.W., Creswell J.D. (2017).

[131] Meisters J., Hoffmann A., Musch J. (2020). Controlling social desirability bias: an experimental investigation of the extended crosswise model. PLoS One.

[132] Saunders M.N. (2015). Handbook of Research Methods on Trust.

[133] Pries K.H., Dunnigan R. (2015).

[134] West B.T., Welch K.B., Galecki A.T. (2022).

[135] Handayani R., Purbasari I., Setiawan D., Ahmadi F., Praswanti R.P. (2021). The role of family education in forming the independent character of students in elementary school. Int. J. Elem. Educ..

[136] Suri D. (2021). Parenting pattern in instilling the character for children from an early age. Jurnal Obsesi: Jurnal Pendidikan Anak Usia Dini.

[137] Salmiati S., Zaman B. (2021). Journal of International Conference Proceedings (JICP).

[138] Grönlund H. (2011). Identity and volunteering intertwined: reflections on the values of young adults. Voluntas Int. J. Voluntary Nonprofit Organ..

[139] Wood R., Berger J., Roberts J. (2017). Corporate Social Responsibility, Sustainability, and Ethical Public Relations.

[140] Brata I.B., Mantra I.B.N., Rai I.B., Wartha I.B.N. (2021). The discourse of informal education: developing children characters during covid-19 pandemic. International Journal of Linguistics and Discourse Analytics.

[141] Rubini R., Chaer M.T. (2021). Children's character education in Javanese muslim families. At-Tarbiyat: Jurnal Pendidikan Islam.

[142] McKinley C., Knipp H., Lilly J. (2022). ‘A learning experience’: disciplinary and parenting practices among Native American families. Child Fam. Soc. Work.

[143] Al-Hassan O.M., De Baz T., Ihmeideh F., Jumiaan I. (2021). Collectivism and individualism: Jordanian mothers' child-rearing values. Int. J. Early Years Educ..

[144] Kress G. (2018). Language and Control.

[145] Hofstede G. (2011). Dimensionalizing cultures: the Hofstede model in context. Online readings in psychology and culture.

[146] Gifford R., Nilsson A. (2014). Personal and social factors that influence pro‐environmental concern and behaviour: a review. Int. J. Psychol..

